# The effect of Robertsonian translocations on the intranuclear positioning of NORs (nucleolar organizing regions) in human sperm cells

**DOI:** 10.1038/s41598-019-38478-x

**Published:** 2019-02-18

**Authors:** Ewa Wiland, Marta Olszewska, Nataliya Huleyuk, Vyacheslav B. Chernykh, Maciej Kurpisz

**Affiliations:** 1 Institute of Human Genetics, Polish Academy of Sciences, Poznan, Poland; 2 Institute of Hereditary Pathology, Ukrainian Academy of Medical Sciences, Lviv, Ukraine; 3 Research Centre for Medical Genetics, Russian Academy of Medical Sciences, Moscow, Russian Federation

## Abstract

Only a few studies have described sperm chromosome intranuclear positioning changes in men with reproductive failure and an incorrect somatic karyotype. We studied the influence of Robertsonian translocations on the acrocentric chromosome positioning in human sperm cells. The basis of the analysis was the localization of NORs (nucleolar organizing regions) in sperm nuclei from three Robertsonian translocation carriers, namely, rob(13;22), rob(13;15) and rob(13;14), with a known meiotic segregation pattern. All three carriers presented with a similar percentage of genetically normal sperm cells (i.e., approximately 40%). To visualize NORs, we performed 2D-FISH with directly labelled probes. We used the linear and radial topologies of the nucleus to analyse the NORs distribution. We found an affected positioning of NORs in each case of the Robertsonian translocations. Moreover, the NORs tended to group, most often in two clusters. Both in Robertsonian carriers and control sperm cells, NORs mostly colocalized in the medial areas of the nuclei. In the case of the Roberstonian carriers, NORs were mostly concentrated in the peripheral part of the medial area, in contrast to control sperm cells in which the distribution was more dispersed towards the internal area.

## Introduction

Robertsonian translocations (ROBs), named after the American biologist W.R.B. Robertson, were first described in 1916 in grasshoppers^1^. Since the first description of ROBs, they have been an object of fascination for geneticists and a subject of numerous and comprehensive studies due to their following characteristics: specificity of the creation of these translocations^2,3^; potential influence on speciation^4^; presence of NOR sequences on chromosomes engaged in the creation of ROBs^5^; and influence on reproductive failure^6,7^.

Robertsonian translocation is a central fusion of the long arms of two acrocentric chromosomes. Acrocentric fusions have been proposed to occur via incomplete homologous or non-homologous recombination between short arm repeats or through the repair of short arm DNA damage, which is corrected by a similar short arm DNA sequence on a nearby non-homologous acrocentric chromosome. In most cases, the regions of breaks are located just above the centromere. A singleton chromosome with two centromeres is then formed (a dicentric chromosome), one of which loses the shape of the centromere and remains inactive. A simultaneously created fragment (acentric) without a centromere is lost during subsequent cell divisions^6,8^. Consequently, in humans, ROB carriers have 45 chromosomes. In most cases, no phenotypic significance is linked to the loss of the short chromosome arms probably because these arms contain only nucleolus organizing regions called NORs^9^. All 10 short arms of acrocentrics have a specific genomic organization, and they share several highly similar blocks of repetitive DNA, including satellite III and beta satellite. The short arms also share NORs, which exist as clusters of approximately 400 copies of 43 kb ribosomal DNA (rDNA) organized in a head-to-tail fashion and localized between centromeric and telomeric heterochromatin^5,10^.

Among five pairs of human acrocentric chromosomes (13, 14, 15, 21 and 22), which can form 10 nonhomologous ROBs, the most commonly occurring ones are rob(13;14) (q10;q10) (67–75%) and rob(14;21) (q10;q10) (approximately 10%)^11–25^. The majority of heterologous ROBs are inherited from a carrier parent.

ROBs are common in man with an incidence of 1 in 1000–1230 births^6,11,14^. Among men with reproductive failure, ROBs occur over 9 times more often, that is, with a frequency greater than 0.8%^6,9^. Problems with fertility in ROB carriers are mostly because during meiosis, the rearranged chromosomes in pachytene form a configuration composed of three chromosomes (trivalent) as a result of paired homologous fragments. In anaphase, these three chromosomes segregate to gametes, resulting in the following segregation types: alternate, adjacent 1, adjacent 2, and 3:0. Fertilization with a spermatozoon created after adjacent or 3:0 segregation leads to trisomy or monosomy in zygotes, and the majority of which are eliminated early^12,15^.

Meiotic segregation patterns have been recognized in about 150 carriers of the following different nonhomologous ROBs: der(13;14), der(13;15), der(13;21), der(13;22), der(14;15), der(14;21), der(14;22), der(15;21), der(15;22) and der(21;22)^17–29^. On average, in most carriers of different nonhomologous ROBs, the frequency of genetically balanced segregants is around 80%, and the numbers of offspring with normal or balanced karyotypes are similar which can indicate a homogenous segregation behaviour of Robertsonian translocations independent of the chromosome pairs involved^16,18,19,25^. Many ROB carriers have oligoasthenoteratozoospermia with varying degree of intensity^16^. Interestingly, carriers with normal sperm parameters or oligoasthenoteratozoospermic (OAT) carriers display similar frequencies of genetic imbalance, suggesting that the segregation pattern and impairment of spermatogenesis are most probably independent processes^19,25^. ROB carriers are often infertile (meaning: no conception), but it happens that they are the sons or the brothers of fertile carriers of the same translocation. It is therefore hard to clearly assess the influence of these translocations on infertility^12,14^.

The aim of this study was to investigate if the presence of Robertsonian translocation interferes with changes of acrocentric chromosome positioning in human sperm cells. The topology and organization of chromosomes in human sperm cells is a subject of numerous studies due to suggestions that intranuclear architecture is of considerable importance for the correct decondensation of chromatin in the male pronucleus^30–32^. In this context, it seems important that changes in the chromosomal topology were detected in men with reproductive failures and correct somatic karyotype^33,34^. However, in our earlies studies, we found changes in the spatial arrangement of chromosomes in sperm cells with chromosome abnormalities, namely, with aneuploidies^35^, with small additional marker chromosome^36^, and with reciprocal translocations^37^. In the studies reported here we investigated the positioning of acrocentric chromosomes in sperm cells from ROBs carriers by analysing the localization of NORs. NORs were chosen as the target region of our interest because the analysis how ROBs affect the positioning of NORs seems to be interesting in view of observed tendency for clustering in control sperm cells in fertile men with normal karyotype^38,39^. To visualize NORs, we performed 2D-FISH with directly labelled probes. The conservation of the distal sequence to rDNA among the five human acrocentric chromosomes provides a unique opportunity to visualize NORs by FISH signals, which are consistent between NORs^40^. We analysed a radial and longitudinal localization of NORs in sperm nuclei from three carriers of different ROBs with known meiotic segregation patterns. We found altered distribution of NORs in each case of the Robertsonian translocation. Such an observation stemmed mostly from the comparison of the localization of NORs in sperm cells from a control donor with normal karyotype vs ROB carriers.

## Results

### Meiotic segregation patterns

The meiotic segregation patterns were examined for the ROB carriers on the basis of ca. 2100–3400 sperm cells counted. As presented in Table 1, in the case of rob(13;15) (R2) and rob(13;14) (R3) carriers, the segregation patterns were similar, and the percentage of sperm cells of the alternate and adjacent segregation types did not significantly differ. The majority of the sperm cells (greater than 75%) were normal and genetically balanced. In the case of rob(13;22) (R1), the percentage of normal sperm cells after the alternate/normal segregation, was similar to results for R2 and R3 (41.5%, 42.9%, and 40.0%, respectively). In turn, the percentage of genetically balanced sperm cells, that is, after the alternate/balanced segregation, and the sum of the unbalanced sperm cells was 10% higher for R1 than for R2 and R3.

**Table 1:** Meiotic segregation patterns in sperm cells of three Robertsonian translocation carriers (R1, R2 and R3) and the number of acrocentric chromosomes with NOR sequence.

Segregation type	No. of chromosomes with NOR	R1 = rob (13; 22)			R2 = rob (13; 15)			R3 = rob (13; 14)		
		sperm genotype*	%	Total %	sperm genotype**	%	Total %	sperm genotype***	%	Total %
2:1 Alternate normal/balanced^a^	5	23	**41.5**	**86.8**	23	**42.9**	**78.4**	23	**40.0**	**75.8**
	3	22, −13, −22, +der (13;22)	**45.3**		22, −13, −15, +der (13;15)	**35.5**		22, −13, −14, +der (13;14)	**35.8**	
2:1 Adjacent-1^**b**^	4	23, −22, +der (13; 22)	2.6	9.9	23, −15, +der (13; 15)	6.5	20.5	23, −14, +der (13; 14)	4.8	18.7
	4	22, −13	1.5		22, −13	3.3		22, −13	2.4	
2:1 Adjacent-2	4	23, −13, +der (13; 22)	3.6		23, −13, +der (13; 15)	5.8		23, −13, +der (13; 14)	6.5	
	4	22, −22	2.2		22, −15	4.9		22, −14	5.0	
3:0 and/or 2n	5	24, +der (13;22)	1.3	1.3	24, +der (13, 15)	0.6	0.6	24, +der (13;14)	2.0	2.0
		∑ unbalanced^c^		11.2	∑ unbalanced		21.1	∑ unbalanced		20.7
		unexplained		2.0	unexplained		0.5	unexplained		3.5

One-way ANOVA was used to compare results (p ≤ 0.01 was considered to be statistically significant).

^a^Value of R1 (total % of Alternate/balanced) was significantly different from the R2, R2/2 and R3 results.

^b^Value of R1 (total % of Adjacent) was significantly different from the R2, R2, R2/2 and R3 results; also, R3 was different from the R2 result.

^**c**^Value of R1 (total % of unbalanced) was significantly different from the R2, R2/2 and R3 results.

As it is known and as detailed in Table 1, the sperm cells from Robertsonian carriers always have 5 acrocentric chromosomes with the NOR sequence after the alternate/normal and after the 3:0 segregation, while the sperm cells after the alternate/balanced segregation have 3 acrocentric chromosomes with the NOR sequence. Sperm cells that are products of adjacent-1 and adjacent-2 segregations have 4 acrocentric chromosomes with NOR sequences.

### Linear localization of NORs in sperm cells

The number of analysed relevant sperm nuclei (i.e., having simultaneously red NORs signal/s and a single green signal of the centromere of chromosome 7) is presented in Table 2. According to the data shown in Table 2, the analyses concerning the intranuclear localization of the NOR sequences (both linear and radial aspects) were performed in 1139 sperm cells (390 + 398 + 351) from three ROB carriers (R1, R2 and R3, respectively) and in 474 sperm cells from a control donor. In these sperm cells, we localized 2205 (720 + 786 + 699, respectively) and 1122 FISH signals originating from the NOR sequences from ROB carriers and control donor, respectively (Table 2). Theoretically speaking, the number of expected NORs recognized as discrete FISH signals observed per cell should be the same as the number of acrocentric chromosomes bearing the NOR sequence in a particular sperm cell. However, the number of discrete FISH signals observed per nucleus in most sperm cells was lower due to acrocentric chromosomes displaying tendencies of colocalization. As a consequence, the sum of observed FISH signals coming from NORs was approximately half the total of acrocentric chromosomes in analysed sperm cells (Table 2).

**Table 2:** The number of sperm cells in which analysis of NORs localization (recognized as FISH signals) was performed. The sum of observed NORs discrete signals versus the number of expected NOR signals if all NOR-bearing chromosomes were independently dispersed.

Carrier	No. of analyzed sperm cells	No. of expected NOR signals = ∑ of chromosomes with NOR sequence	∑ of observed NORs signals	% of expected NORs signals
**R1 rob(13;22)**	**390**	1494	720	**48%**
**R2 rob(13;15)**	**398**	1604	786	**49%**
**R3 rob(13;14)**	**351**	1342	699	**52%**
∑/mean value	1139/380	4440/1480	2205/735	50%
**Control**	**474**	2370	1122	**47%**

**Figure 1:**
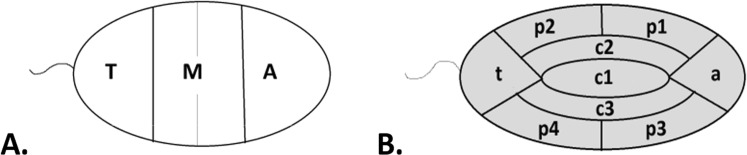
A scheme of the sperm cell nucleus areas used for the localization of FISH signals from NORs (nuclear organizing regions). (**A**) Linear division. The division of the sperm cell nucleus into three longitudinal areas was marked as follows: A = apical, closer to the acrosome; M = medial; and T = area close to tail. (**B**) Radial division. The division of the sperm cell nucleus into nine radial areas was marked as follows: (c1) = central; (c2 + c3) = internal; (p1 + p3) = peripheral, near the apical area; (p2 + p4) = peripheral, near the tail area; (a) = apical area; and (t) = tail area.

The linear organization of NORs was assessed using the scheme depicted in Fig. 1A. Table 3 shows the results of distribution of discrete FISH signals visualized for NORs in three linear areas (A), (M) and (T). In the sperm cells of carriers R1, R2 and R3 as well as in the sperm cells of the Control, the majority of NORs (i.e., 54.0%, 56.7%, 51.1% and 58.4%, respectively) were localized in the (M) area, while the minority of NORs were localized in the (T) area (12.0%, 11.4%, 16.4% and 15.0%, respectively). Significant differences concerned mostly the (A) area. In all carriers, i.e., R1, R2 and R3, considerably more NORs appeared in the (A) area than in the Control sperm cells (34.0%, 31.9% and 32.5% vs. 26.6%, respectively). The differences between the carriers concerned only values in areas (M) and (T), which differed for R3 from the values for R1 and for R2 (see Table 3).

**Table 3:** Linear localization of NORs in sperm cells nuclei. Percentage (%) of NORs (recognized as FISH signals) in marked linear areas A, M, and T as defined in Fig. 1A.

Carrier	Linear areas of nucleus^•^			Sperm cells with NORs localized only in the M area	
	A	M	T		
	% of NORs			% of sperm cells	% of NORs
**R1 rob(13;22)**	34.0*	54.0* ^**H**^	**12.0** ^**L**^	**43.9**	40.1*
Number of analyzed objects	245/720	389/720	86/720	171/390	289/720
∑ of all analyzed NORs signals = 720 ∑ of all analyzed sperm cells = 390					
**R2 rob(13;15)**	31.9*	**56.7** ^**H**^	11.4* ^**L**^	60.1*	56.9*
Number of analyzed objects	251/786	446/786	89/786	238/398	449/786
∑ of all analyzed NORs signals =786 ∑ of all analyzed sperm cells = 398					
**R3 rob(13;14)**	32.5*	51.1* ^**1H**^	**16.4** ^**1L**^	56.4* ^**2**^	54.2*^2^
Number of analyzed objects	227/699	357/699	115/699	198/351	375/699
∑ of all analyzed NORs signals = 699 ∑ of all analyzed sperm cells = 351					
**Control**	**26.6**	**58.4** ^**H**^	**15.0** ^**L**^	**47.5**	45.5
Number of analyzed objects	299/1122	655/1122	168/1122	225/474	510/1122
∑ of all analyzed NORs signals = 1122 ∑ of all analyzed sperm cells = 474					

^•^Linear areas according to the scheme in Fig. 1A. One-way ANOVA was used to compare results (p ≤ 0.01 was considered to be statistically significant). *Values significantly different from mean control value C.

^a^Significantly different from the R1 and R2 results.

^b^Significantly different from the R1 result.

^L^Lowest values between areas (A, M and T).

^H^Highest values between areas (A, M and T).

During the analysis of linear distribution of NORs signals, it was also noted that in a significant percentage of the sperm cells (from approximately 44% to 60%), all signals were located only in the (M) area. Table 3 also presents individual results for carriers R1, R2, R3 and the Control. Compared to the Control value (47.5%), the result for R1 (43.9%) was similar, whereas significant differences were found for R2 and R3 (60.1% and 56.4%, respectively).

Table 4 shows in detail the results indicating the NOR tendency for clustering. In the case of Control sperm cells, the presence of five discrete NOR signals, which would be expected if all the acrocentric chromosomes were randomly or independently localized, was only observed in 0.4% of nuclei. The presence of four NOR signals was small (9.4%). A single NOR signal was observed in 16.6% of Control sperm cells. In most Control sperm cells, NORs formed two (41.4%) or three (32.1%) discrete signals. In the case of R1, R2 and R3 carriers, sperm cells with five discrete NOR signals were not observed, and sperm cells with four signals were rare (approximately 4%). In all three R1, R2, and R3 carriers, the percentage of sperm cells with two separate NOR signals (38.5%, 43.2% and 44.4%, respectively) was similar to Control sperm cells (41,4%). Significant differences occurred among R1, R2 and R3 carrier sperm cells with three NOR signals, and the number of cells with three NOR signals was less than that in Control sperm cells (23.0%, 23.0% and 23.1%, respectively). The number of sperm cells from R1, R2 and R3 carriers with one NOR signal, was significantly more than that of the control (34.4%, 29.6% and 28.2%, respectively). Thus, the highest percentage of sperm cells in R1, R2, R3 and Control (about 40%) was sperm cells with two NOR signals (Table 4). Moreover, in the case of R1, R2, and R3 carriers, the sperm cells with one or two NOR signals jointly constituted as many as 73% but only 58% in the Control sperm cells. In the case of the Control, however, the majority (73.5%) of sperm cells jointly consisted of sperm cells with two or three NOR signals.

**Table 4:** Clustering of NORs in sperm cells. Percentage (%) of sperm cells in which NORs (recognized as FISH signals) form one, two, three, four or five clusters.

Number of NORs clusters	1	2	∑(1 + 2)	3	4	5
Carrier	% of sperm cells					
**R1 rob(13;22)**	34.4* ^a^	**38.5** ^**Hb**^	72.9*	23.0* ^**,A**^	4.1* ^**L**^	0.0
Numer of sperm cells	134/390	150/390	284/390	90/390	16/390	0.0
∑ of analyzed sperm cells = 390 ∑ of analyzed NORs signals = 720						
**R2 rob(13;15)**	29.6*	**43.2** ^**H**^	72.8*	23.0* ^**A**^	4.2* ^**L**^	0.0
Numer of sperm cells	118/398	172/398	290/398	91/398	17/398	0.0
∑ of analyzed sperm cells = 398 ∑ of analyzed NORs signals = 786						
**R3 rob(13;14)**	28.2*	**44.4** ^**H**^	72.6*	23.1* ^**A**^	4.3* ^**L**^	0.0
Numer of sperm cells	99/351	156/351	255/351	81/351	15/351	0.0
∑ of analyzed sperm cells = 351 ∑ of analyzed NORs signals = 699						
**Control**	**16.6**	**41.4** ^**H**^	**58.0**	**32.1**	**9.4**	**0.4** ^**L**^
Numer of sperm cells	79/474	197/474	276/474	153/474	45/474	2/474
∑ of analyzed sperm cells = 474 ∑ of analyzed NORs signals = 1122						

One-way ANOVA was used to compare results (p ≤ 0.01 was considered to be statistically significant).

*Values significantly higher than the Control value (p < 0.01).

^a^Significantly different from the R2 and R3 results.

^**b**^Significantly different from the R2 and R3 results.

^H^Significantly higher than the remaining results.

^L^Significantly lower than the remaining results.

^A^Significantly different from the remaining results.

Similar results indicating the tendency of NOR signals to group were also found in these sperm cells, in which all signals were found only in the (M) area (Table 5) (also see right side of Table 3). In most Control sperm cells, NORs formed two (45.3%) or three (27.6%) discrete signals, but NORs in most sperm cells from R1, R2 and R3 formed two (37.4%, 53.9% and 44.0%, respectively) or one cluster (46.8%, 28.9% and 33.3%, respectively) (Table 5). Differences both in relation to Control values and among R1, R2 and R3 were statistically significant.

**Table 5:** Clustering of NORs in the case of sperm cells in which all NORs (recognized as FISH signals) were located only in the M (medial) area of the sperm cell nucleus (M area marked in the scheme in Fig. 1A).

Number of NORs cluster only in M area of nucleus	1	2	3	4 or 5
Carrier	% of sperm cells			
**R1 rob(13;22)**	46.8*	37.4*	15.8*	0.0*
Number of sperm cells with NORs signals only in M = 171 = **43.9%** ∑ of all analyzed sperm cells** = **390	80/171	64/171	27/171	0/171
**R2 rob(13;15)**	28.9*	53.9*	17.2*	0.0*
Number of sperm cells with NORs signals only in M = 238 = **60.1%** ∑ of all analyzed sperm cells** = **398	69/239	129/239	41/239	0/239
**R3 rob(13;14)**	33.3* ^a^	**44.0** ^b^	22.7* ^c^	0.0*
Number of sperm cells with NORs signals only in M = 198 = **56.4%** ∑ of all analyzed sperm cells** = **351	66/198	87/198	45/198	45/198
**Control**	**18.7**	**45.3**	**27.6**	**8.4**
Number of sperm cells with NORs signals only in M = 225** = 47.5%** ∑ of all analyzed sperm cells** = **474	42/225	102/225	62/225	19/225

One-way ANOVA was used to compare results (p ≤ 0.01 was considered to be statistically significant).

*Values significantly different from Control value (p < 0.01).

^a^All of the values of R1, R2 and R3 were significantly different.

^b^All of the values of R1, R2 and R3 were significantly different.

^c^All of the values of R1, R2 and R3 were significantly different.

**Figure 2:**
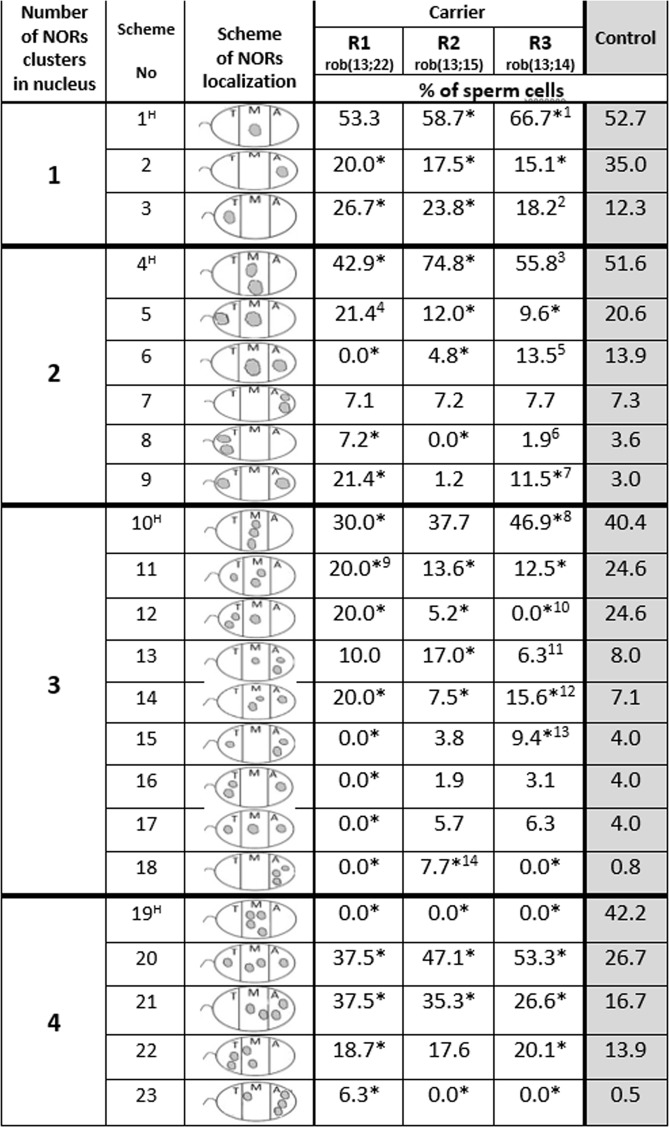
Percentage (%) of sperm cells with NORs localizations (recognized as FISH signals) according to schemes No. 1 – 23 (for representative images of FISH, see Suppl. Fig. S1). One-way ANOVA was used to compare results (values p ≤ 0.01 was considered to be significantly different). *Values were significantly different from the Control value (p < 0.01). ^H^Nr of the scheme, the frequency of sperm cells was the highest (p < 0.001) in R1, R2, R3 and Control. ^**1**^Significantly different from the R1 and R2 results. ^**2**^Significantly different from the R1 result. ^3^All of the values of R1, R2 and R3 were significantly different. ^**4**^Significantly different from the R2 and R3 results. ^5^All values R1, R2 and R3 were significantly different. ^**6**^Significantly different from R1 result. ^7^All of the values of R1, R2 and R3 were significantly different. ^**8**^Significantly different from the R1 and R3 results. ^**9**^Significantly different from the R2 and R3 results. ^10^All of the values the R1, R2 and R3 were significantly different. ^11^All of the values of R1, R2 and R3 were significantly different. ^12^All of the values of R1, R2 and R3 were significantly different. ^13^Significantly different from the R1 and R2 results. ^14^Significantly different from the R1 and R2 results.

Figure 2 shows schemes of different NOR localizations in nuclei (recognized as red FISH signals) (For their corresponding representative images see Suppl. Fig. S1). Simultaneously, the percentages of sperm cells in which NORs were found in particular localizations in R1, R2, R3 and the Control are also shown (for total amount of sperm cells with given numbers of NOR clusters see Table 4). As a criterion for the order of schemes (i.e., number 1–23), we assumed the decreasing percentages of the relevant sperm cells in the Control. A considerable part of the detailed results for R1, R2 and R3 was different in relation to Control and/or in relation to one another. For R1, R2 and R3, there were few spermatozoa with four NOR signals (see Table 4 and scheme numbers 19–23 in Fig. 2). These differences may be attributed to the fact that the number of the analysed NOR signals was higher in the case of the Control (see Tables 2 and 4). However, despite the differences, the interesting similarity of the results for R1, R2, R3 and the Control was noticeable because in the case of one, two, or three clusters of NORs, the most sperm cells were compatible with the same scheme, that is, number 1, 4, and 10, respectively. Scheme numbers 1, 4 and 10 concerned signals localization only in the medial (M) area. With respect to the Control, the largest difference existed in sperm cells with four NOR signals. In the case of R1, R2 and R3, no sperm cells in which all four signals were concentrated only in the M area (i.e., according to scheme No 19) were found.

### Radial localization of NORs in sperm cells

**Figure 3:**
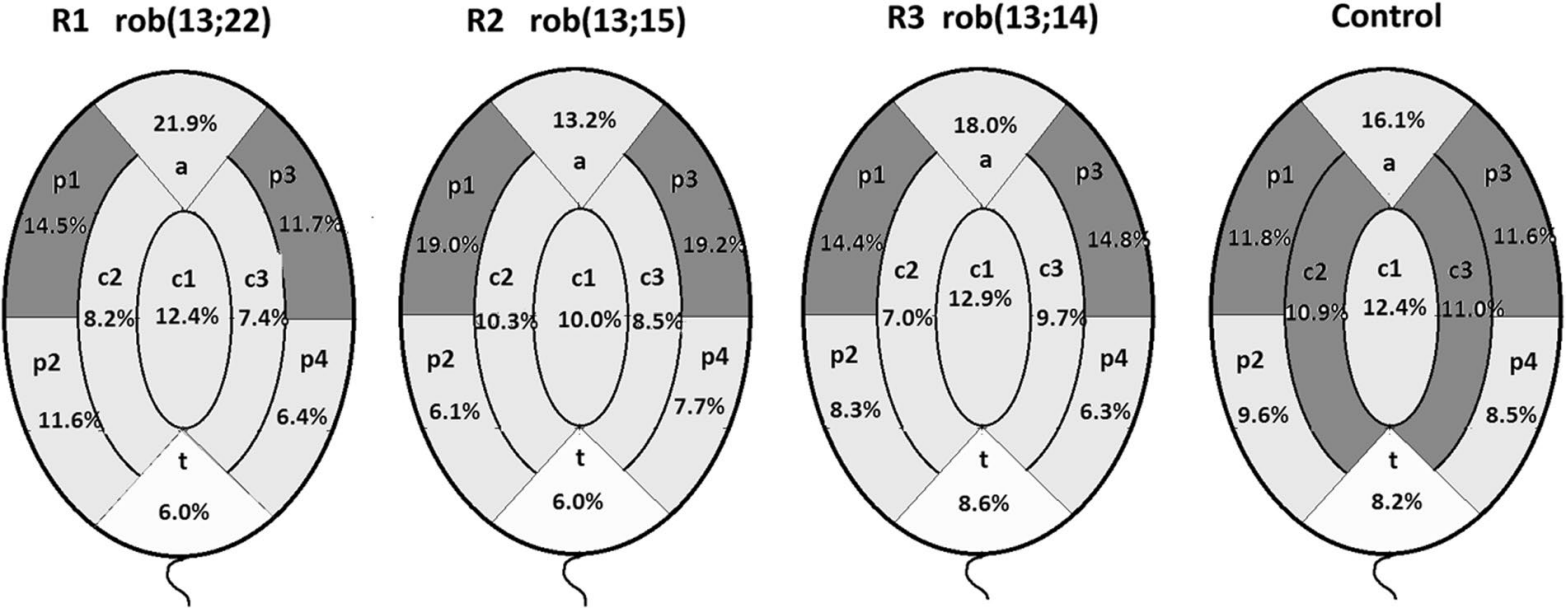
Illustration of the results of the radial localization of NORs in sperm cells from Robertsonian translocation carriers (R1, R2 and R3) and the Control. Values based on the data from Table 6. Values in dark grey mark areas, including (p1 + p3) and in the case of the Control also (c2 + c3), were significantly higher, while the values in the white areas (t) were found to be the lowest (data in Table 6). The division of the sperm cell nucleus into radial areas according to the scheme in Fig. 1B.

The radial organization of NORs was assessed using the scheme depicted in Fig. 1B. Table 6 shows the results of distribution of discrete FISH signals visualized for NORs in nine radial areas. The illustration of these results is shown in Fig. 3 (for more detailed data see Suppl. Fig. S2). The results in the depicted areas are analysed as a sum, i.e., p1 + p3, p2 + p4 and c2 + c3, because both sides of the sperm nucleus, namely front and back, could not be distinguished using the fluorescent microscope in this study. Due to the flattened shape of the sperm cells, fixed nuclei were arranged on the slide randomly on the “front” or the “back” side (see Fig. 1B). Therefore, the “mirror reflection effect” must be considered in the analysis of the results of intranuclear topology of FISH signals.

**Table 6:** Radial localization of NORs in sperm cells nuclei. Percentage (%) of NORs (recognized as FISH signals) in radial areas (a), (c1), (c2 + c3), (p1 + p3), (p2 + p4) and (t) as defined in Fig. 1B and depicted in Fig. 2.

Radial areas in sperm cell^•^	a	c1	c2 + c3	p1 + p3	p2 + p4	t
**Carrier**	**% of NORs**					
**R1 rob(13;22)**	21.9* ^**A**^	**12.4** ^**B**^	15.6*	**26.2** ^**H**^	**17.9** ^**C**^	**6.0** ^**L**^
Number of NORs signals	158/720	89/720	112/720	189/720	129/720	43/720
∑ of NORs signals = 720						
∑ of sperm cells = 390						
**R2 rob(13;15)**	**13.2** ^**1,D**^	**10.0**	**18.8** ^**E**^	38.2* ^2,H^	13.8* ^3^	**6.0** ^**L**^
Number of NORs signals	104/786	78/786	148/786	300/786	108/786	48/786
∑ of NORs signals = 786						
∑ of sperm cells = 398						
**R3 rob(13;14)**	**18.0** ^**F**^	**12.9** ^**G**^	16.7*	29.2* ^**H**^	**14.6**	**8.6** ^**L**^
Number of NORs signals	126/699	90/699	117/699	204/699	102/699	60/699
∑ of NORs signals = 699						
∑ of sperm cells = 351						
**Control**	**16.1** ^**I**^	**12.4** ^**J**^	**21.9** ^**K**^	**23.4** ^**M**^	**18.0**	**8.2** ^**L**^
Number of NORs signals	181/1122	139/1122	246/1122	262/1122	201/1122	92/1122
∑ of NORs signals = 1122						
∑ of sperm cells = 474						

^•^Radial areas according to the scheme shown in Fig. 1B. Radial areas were labeled as follows: (c1) = central; (c2 + c3) = internal,(p1 + p3) = peripheral, near the apical area, (p2 + p4) peripheral, near the tail area, (a) = apical area and (t) = tail area. One-way ANOVA was used to compare results (p ≤ 0.01 was considered to be significantly different). *Values significantly different from Control value (p < 0.01).

^H^In cases when the R1, R2 and R3 values in the (p1 + p3) area were highest.

^L^In cases when the of R1, R2, R3 and Control values in the t area were highest.

^**A**^Value not different only from the (p2 + p4) value.

^**B**^Value not different only from the (c2 + c3) value.

^C^Value not different only from the (c2 + c3) value

^D^Values of (a), (c1) and (p2 + p4) were not different.

^E^Value of (c2 + c3) was different from the other areas.

^**F**^Value not different only from the (c2 + c3) and (p2 + p4) values.

^**G**^Value not different only from the (p2 + p4) values.

^**I**^Value not different only from the (c1) and (p2 + p4) values.

^**J**^Value not different only from the (a) value.

^**K**^Value not different only from the (p1 + p3) and (p2 + p4) values.

^**M**^Value not different only from the (c2 + c3) value.

^a^Significantly different from the R1 and R3 values.

^b^Significantly different from the R1 and R3 values.

^c^Significantly different from the R1 value.

Regardless of the significant differences in the results among R1, R2 and R3, the highest percentage of discrete NORs signals (i.e., 26.2%, 38.2%, and 29.2%, respectively) in the sperm cells of all three ROBs were localized in the (p1 + p3) area, while the lowest were localized in the (t) area (6.0%, 6.0% and 8.6%, respectively) (Table 6).

In the case of Control sperm cells, the lowest percent of discrete NORs signals was also found in the (t) area. However, considerably more NORs appeared both in the (p1 + p3) and in (c2 + c3) areas (Fig. 3, Table 6 data). These data indicated a more scattered dispersion of NOR locations in the Control sperm nuclei compared to the locations in sperm nuclei from R1, R2 and R3.

Correlated to the data presented in Fig. 3 (for more details see Suppl. Fig. S2C), the results in radial area p1 were similar to the results in p3 in every studied case (R1, R2, R3 and Control). Hypothetically, such symmetry could indicate a lack of the tendency for preferential localization of NORs on one side of the sperm nucleus or the other (i.e., p1 or p3). However, due to the inability to differentiate between the “back” and the “front” side of fixed sperm nuclei, we could not rule out that the existence of such a preference might remain undetected.

**Figure 4:**
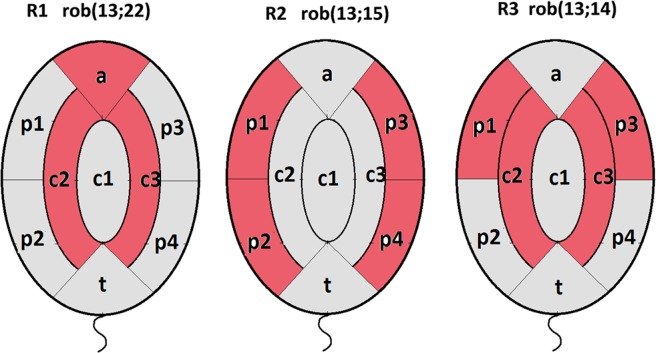
Illustration of the differences in the radial localization of NORs in sperm cells from Robertsonian translocation carriers (R1, R2 and R3) compared to the Control (values based on the data from Table 6). The red background illustrates areas with results that were significantly different from the Control values (data in Table 6). The division of the sperm cell nucleus into radial areas according to the scheme shown in Fig. 1B.

The significant differences in the individual results of R1, R2 and R3 (Table 6) compared to the Control are shown in Fig. 4. For each of the carriers R1, R2, and R3, the differences compared to the Control are marked across different areas, which indirectly illustrate the differences between carriers. Significant differences were not present only in the (c1) and (t) areas.

## Discussion

The relationship between the human sperm intranuclear architecture and the sperm cell function is not fully recognized^30,41^. However, there is a generally accepted view that changes in sperm chromosome organization may be critical for pronuclear chromatin remodelling, which in consequence may disturb the transmission of paternal chromosomal information to the zygote^31^.

Sperm cell chromosomes are approximately 6 times more tightly condensed when compared to the chromosomes of somatic cells^42,43^, and sperm FISH studies have shown that whole chromosomes are preferentially ordered along the head-tail axis and occupy individual territories^33,43–46^. Several concepts have been presented for the model of intranuclear architecture of human sperm cells. First a structural layout of the sperm DNA/chromatin has been presented in the so-called donut-loop model^32^. The so-called hairpin-loop model, the model based on double normalized, preferential 2D-FISH data, assumes that heterochromatin of chromosome centromeres form an inner one to three clusters in the interior area of the nucleus called the chromocentre. At the same time, chromosome telomeres have been preferentially located at the nuclear periphery of the nucleus forming dimers and tetramers^43,44,46^. More recent studies performed using a confocal microscope (3D-FISH) have shed additional light on the knowledge of topology of sperm chromosomes^33,39,45^. Confocal microscope data have suggested that sperm nuclei centromeres cluster to form multiple chromocentres (with an average of 7 chromocentres observed per cell) that display a more segmented organization occupying discrete locations with preferential (92%) intermediate and peripheral localizations. Moreover, the organization of dimers or tetramers of telomeres is more segmental with a significant proportion of telomeres clustering to form a “belt” in the mid part of the sperm nucleus^39^.

The results of the research performed so far indicate that all the factors that have been analysed with respect to their negative influence on spermatogenesis (e.g., chromosomal reciprocal translocations and high DNA fragmentation) can disrupt the architecture of sperm nucleus^33,35–37,47,48^.

Having analysed the dispersions of NORs in the reported studies (recognized as 2D-FISH discrete signals), we found changes in the nuclear architecture in spermatozoa from Robertsonian translocation carriers in comparison to control sperm cells.

To date, only few studies have examined the effects of chromosomes with Robertsonian translocations on nuclear organization in mammalian germ cells^49–52^. In mouse germ cells from heterozygous carriers of multiple Robertsonian translocations, Garagna *et al*.^49^ showed an altered nuclear organization in spermatocytes and spermatids. Berríos *et al*.^50^ showed that meiotic architecture of mouse spermatocytes with Robertsonian chromosomes is prone to modification by chromosomal rearrangements. Moreover, the results of Solé *et al*.^51^ provided the first evidence of a chromosome territorial alteration in the presence of a Robertsonian translocation in metaphase I of human spermatocytes. In turn, the results presented by Acloque *et al*.^52^ showed that boar Robertsonian translocation t(13;17) does not significantly alter sperm nucleus architecture, but they suggested that centromere remodelling after chromosome fusion locally impacts chromosome positioning^52^.

In the present study, the topology of NORs in sperm cells from three Robertsonian carriers, namely rob(13;22), rob(13;15) and rob(13;14), was analysed. The common feature of these translocations was that they were related to chromosome 13, and all three carriers had the same percentage of normal sperm cells (i.e., approximately 40%). Moreover, in each case, the minority of the sperm cells (i.e., less than 25%) were genetically unbalanced (Table 1). These results were in line with other studies, in which a majority (72.2–96.6%) of sperm originated by alternate segregation^17–29^.

We found an altered distribution of NORs in each case of examined ROBs. Such an observation stems mostly from the comparison of the dispersion of NOR signals in the Control sperm cells with normal karyotype (Table 3 for linear and Table 6 with Fig. 4 for radial distribution). The difference with respect to the Control mainly concerned the higher percentage (%) of discrete NOR signals found in the apical area (Table 3). However, in the sperm cells of ROB carriers and of the Control, the majority of NORs (i.e., greater than 50%) were localized in the (M) area (Table 3).

Interestingly, we observed a tendency of NOR clustering and colocalization in particular areas of the nucleus both in ROB carriers and in the Control. Both in ROB carriers and Control sperm cells, we found out that NORs showed a tendency towards two discrete FISH signals (Table 4). In the case of the sperm cells from ROB carriers, one or two signals were observed in as many as 72% of the sperm cells. This percentage was significantly more than in the Control (58%), which can indicate a lower tendency towards grouping (Table 4). In the case of clusters formed by two or three acrocentric chromosomes (73.5% of Control sperm cells), it is unknown whether they were preferentially formed by the same chromosomes in different sperm cells or if the grouping was random in individual sperm cells. Unfortunately, the applied FISH probes did not allow us to make such a distinction. NORs are located on the short arms of the five acrocentric chromosomes between centromeric and telomeric heterochromatin. Because the sequences in this region are conserved, the FISH signals are consistent among different NORs^40^.

In addition, our research showed that both in sperm cells of ROB carriers and the Control, the discrete NORs signals were localized mostly in the medial area of the nucleus (Table 3 and Fig. 2). However, in the case of the ROB carriers, the localization mostly appeared in the peripheral part of the medial area in contrast to Control sperm cells in which the distribution was more dispersed towards the internal area (Fig. 3, Table 6).

In contrast to transcriptionally non-active haploid sperm cells, there are 10 NOR sequences in most diploid human cells that contribute to the formation of nucleoli which are the sites of ribosome biogenesis^10,16^. NORs coalesce to form mostly one to several nucleoli which disintegrated during mitosis^40,51,53,54^ (for the representative image of 2D-FISH of NORs in diploid cell see Suppl. Fig. S3). The localization of nucleoli has been examined also during mammalian spermatogenesis. NORs have been observed using silver staining during the entire period of the meiotic prophase up to methaphase I^55,56^. It has been suggested that the association among NOR-bearing chromosomes mainly depends on the presence of constitutive heterochromatin, which is a key element in the nuclear architecture of spermatocytes^52^. Moreover, it has been shown that the round spermatid contains a distinct nucleolus, indicating that some, if not all, of NORs are clustered together in a structural unit^57^. Because later stages of the spermatid do not exhibit a predominant nucleolus, it has been suggested that the structure has begun to segregate into separate chromosomes^58^.

The first evidence of the tendency of NORs to colocalize in mature human sperm cells from fertile control men was suggested in a report of Gurevitz *et al*.^38^. The analysis of dispersion was narrowed only to sperm cells showing five discrete FISH signals, which limited the comparison with our data^38^. The results demonstrated that the centromeres of the five acrocentric chromosomes are positioned close to each other as reflected by their non-random proximity but that their localization is not stable among the different sperm cell nuclei.

In Syrian hamster sperm cells, which have five NOR-bearing chromosomes, sperm nuclei are most commonly (64%) observed to have four or five irregularly distributed distinct FISH signals (using 28S rRNA gene as a probe)^58^. A clear tendency for colocalization at the equatorial region of the nucleus has also been observed in equine sperm cells^59^.

A tendency of non-random colocalization of human acrocentric chromosomes in the central area within the sperm nucleus has also been noted by a 3D-FISH^45^. Ioannou *et al*.^39^ showed that the radial and longitudinal topology of NORs is non-randomly organized and highly reproducible among the sperm cells from 10 fertile control men enrolled in the study. In these studies, a single or five distinct NOR signals were rarely observed in sperm cells (<10%), but NORs predominantly formed three to four discrete signals (>63%)^39^. In our research, NORs in sperm cells from the fertile Control predominantly formed two or three NORs signals (73.5%). The above data together can suggests that in control sperm cells with “healthy nuclear state” NORs predominantly form two to four clusters relatively evenly dispersed in the medial/internal area. In the studies performed so far, it is not possible to determine if clusters are preferentially form by NORs located on these same acrocentric chromosomes^39^. It can only be speculated that the tendency of NORs towards cluster formation depends mainly on the presence of constitutive heterochromatin, which embeds rDNAs, suggesting that it is probably not chromosome specific.

The results of the present studies showed that similar to the existence of reciprocal translocations^37^, the existence of Robertsonian translocations affects the sperm chromosome topology. It is known that there are often infertility problems among ROBs carriers and it is assumed that these problems stem from meiotic disorders. It is therefore difficult to assess the potential implications of the observed repositioning of NORs on the sperm cell function at the current state of the art. Still, an open question remains of how the nucleolus initially forms *de novo* during early embryogenesis in humans^5^. Some inconsistencies among the previously published data undoubtedly complicate the interpretation^30,31,39,44^. Some authors suggest that even if spermatogenesis is compromised, the reorganization of the nuclear architecture is a robust process and topological differences between infertile and fertile males are rather modest^48^. By contrast, the differences in the localization of sperm chromosomes between sperm cells of fertile and infertile men indirectly confirm the hypothesis that changes in the organization of sperm chromosomes may disrupt the transmission of paternal chromosomal information to the zygote^30,31^. However, it must be noted that the conclusions regarding the repositioning of the chromosomes of infertile sperm cells are based on significant differences between the investigated parameters with respect to control sperm cells. By contrast, the observations of the target loci in individual sperm cells (also in the control) (2D or 3D-FISH) show such a great variation that we can put a hypothesis that the model(s) layout^31,39,44,60^ is not directly reflected in any single sperm cell. To paraphrase, sperm cells apparently do not know that they should follow a particular layout.

In summary, according to our knowledge, this is the first study based on human sperm cells, whose goal was to investigate if ROBs carriers show changes in the localization of sperm cell acrocentric chromosomes. On the basis of the NORs analysis in three different ROBs we can only suggest the following: (1) in ROBs carriers there can be perturbations in nuclear organization of sperm acrocentrics, (2) in the case of NORs clusters, there is a tendency to repositioning toward the periphery of the nucleus, and (3) between the carriers, there can be individual differences in nuclear spatial arrangement of the loci that we examined. There are two probable sources of these differences. First, different acrocentric chromosomes are involved in different ROBs. Second, in the sperm cells of over half of ROB carriers, there is an aneuploidy of chromosomes that are not involved in translocations – in our earlier study we showed the presence of dislocated centromere positions in human sperm cells with aneuploidies^35^. However, the answer to the question whether carriers of different ROBs show particular topological features will require future studies on a larger group of carriers of different ROBs.

## Materials and Methods

### Male participants

This study was approved by the Local Bioethical Committee at the Poznan University of Medical Sciences, Poland, and informed consent was obtained from all subjects. We should like also to confirm that all experiments were performed in accordance with relevant guidelines and regulations. The collected patient group consisted of three Robertsonian carriers (ROBs) with reproductive failures. The carriers aged between 30 to 35 years were selected for the study after attending infertility clinics due to the lack of conception over a 5-year period. Two patients were diagnosed as rob(13;22) and rob(13;14) in Lviv (N. Huleyuk), and one patient from Moscow (V. Chernykh) was diagnosed as a carrier of rob(13;15). The Robertsonian carriers were coded as follows: R1 = rob(13;22), R2 = rob(13;15), and R3 = rob(13;14). The analysis of meiotic segregation patters and analysis of localization of NORs were performed in sperm nuclei of carriers R1, R2 and R3. Additionally, analyses of NORs were performed in control sperm nuclei. Sperm cells from a normozoospermic volunteer, who has proven fertility, a normal karyotype, and has attended the Andrology Outpatient in Poznań (M. Kurpisz), served as the control.

### Semen collection and processing

Semen samples were obtained by masturbation after 3–5 days of sexual abstinence. After liquefaction of ejaculate (at room temperature), routine semen analyses were performed according to the World Health Organization 2010 criteria^61^. General semen assessment of carriers R1, R2, R3 and the control volunteer is presented in Suppl. Table S1. Sperm cells were separated from seminal plasma by centrifugation at 600 g for 8 minutes. An aliquot of sperm suspension was washed three times in phosphate buffer saline (PBS; pH 7.4) and processed for FISH analysis.

### FISH (fluorescence *in situ* hybridization) procedure and DNA probes

After a PBS wash, sperm cells were fixed with a fresh, cold fixative solution (methanol: acetic acid 3:1 v/v, −20 °C) for 20 min. After three rinses with the fixative, sperm samples were spread onto slides and air-dried. Prior to FISH reactions, mild decondensation of nuclei was performed, which is a prerequisite for sperm nuclei analysis. Slides were washed two times in PBS and plunged into a solution of 10 mM dithiothreitol (DTT, Sigma, St. Louis, MO, USA) in 0.1 M Tris-HCL (pH 8.5) for 5–10 min in 43 °C. The decondensation of sperm nuclei was verified under a phase-contrast microscope. Under this mild procedure, the sperm cells had a well-defined boundary, and the sperm tails were attached to their heads and nuclei were minimally swollen^47^. Importantly, it has been shown previously that this procedure does not alter the morphology and nuclear topology^38,62^. The slides were rinsed twice in 2x SSC, dehydrated in an ethanol dilution from 70% to 100% and air-dried.

FISH experiments for analysis of meiotic segregation patterns were performed using red or green directly labelled probes from Cytocell Technologies Ltd. (Cambridge, UK) according to the manufacturer’s guideline. This study used 13 wcp red (whole chromosome paint, Texas Red) and 14, 15 or 22 wcp green (whole chromosome paint, FITC) probes. The use of wcp probes required only a moderate sperm decondensation to provide high hybridization efficiency. The wcp method allowed the distinguishment of both normal spermatozoa (with two clearly separated signals of different colour) and balanced spermatozoa (with two signals of different colour coupled one with the other). FISH experiments for the identification of nuclear organizing regions (NORs) were performed using directly labelled Acro-P-Arms NOR red probes from Kreatech Biotechnology B.V. (distributed by Leica Biosystems, UE). Simultaneously, we used an alpha-satellite centromeric chromosome 7 green probe (FITC) to control hybridization efficiency. We utilized the manufacturer’s standard protocol. The hybridization mixture (probes plus hybridization buffer) was applied to the slide, covered with a coverslip and sealed with Fixogum. The probes and cellular DNA were co-denatured simultaneously for 5 min at 75 °C.

Hybridization was performed overnight in a moist chamber at 37 °C. Post-hybridization washes of slides were performed by incubation without agitation for 2 min in 0.4x SCC at 72 °C followed by incubation for 30 sec in 2x SCC/0.5% Tween 20 at 24 °C. Slides were air-dried for several minutes at RT, and 15 µl of DAPI/antifade counterstain was applied. Hybridization signals were observed using the Olympus Bx41 microscope fitted with filters for DAPI/FITC/TEXAS Red and an oil immersed objective 100 × (1.25 NA). Images were captured and archived using a CCD camera and ISIS (MetaSystems, Germany) software. The overall efficiency of FISH was approximately 98–99%. Sperm nuclei were scored according to standard, published criteria^37^. The analysis criterion was not the size of the FISH signal/s but the number of clearly separated, distinguished signals.

### Determination of NOR positioning in sperm nuclei of Robertsonian carriers and control volunteers

To determine the intranuclear position of NORs in examined sperm nuclei, we applied the graphic schemes shown in Fig. 1A,B for the longitudinal and radial localizations, respectively. The schemes included typical ellipsoid shape of the sperm cell where the length to width ratio is ca. 2. The linear analysis was performed as per previous studies for 2D FISH^37^. As presented in Fig. 1A, three contractual linear areas based on the tail-head distance, namely A, M and T, within the nucleus were designated. The distribution of each FISH signal was assigned to a selected area. In the case of Fig. 1B, nine contractual radial areas, namely a, c1, c2, c3, p1, p2, p3, p4, and t, were designated.

### Statistical analysis

The data were analysed using STATISTICA version 7.0 (StatSoft, Tulsa, OK, USA.). One-way ANOVA test was utilized to compare results between patients and control values, and the Chi-squared test was used to compare the frequencies of adjacent and alternate segregation patterns as well as to compare the distribution of signals in nuclear areas. Statistically significant differences were considered as p-value ≤ 0.01.

## Supplementary information

Supplementary Info
