# Supplementary material for: The effect of Robertsonian translocations on the intranuclear positioning of NORs (nucleolar organizing regions) in human sperm cells
Source: Sci Rep. 2019 Feb 18;9:2213. doi: 10.1038/s41598-019-38478-x (PMC6379386; doi:10.1038/s41598-019-38478-x)
Supplement: Supplementary file 1 — Supplementary Info [file 41598_2019_38478_MOESM1_ESM.pdf]

The effect of Robertsonian translocations on the intranuclear positioning of NORs (nucleolar organizing regions) in human sperm cells

Ewa Wiland[1], Marta Olszewska[1], Nataliya Huleyuk[2], Vyacheslav B. Chernykh[3] & Maciej Kurpisz[1]*

[1]Institute of Human Genetics, Polish Academy of Sciences, Poznan, Poland

[2]Institute of Hereditary Pathology, Ukrainian Academy of Medical Sciences, Lviv, Ukraine

[3]Research Centre for Medical Genetics, Russian Academy of Medical Sciences, Moscow, Russian Federation.

*Corresponding author: M.K. e-mail: kurpimac@man.poznan.pl;

first author email: ewila@man.poznan.pl).

Running Title: Topology of NORs in spermatozoa of Robertsonian carriers.

Key words: NOR distribution in human sperm cells, Robertsonian translocation, meiotic segregation pattern.

Supplementary Table S1. Semen assessment of three Robertsonian translocation carriers (R1, R2 and R3) and the control volunteer with normal karyotype.

No.	Karyotype	Spermiogram (according to WHO, 2010)[63]	Reproductive history
R1	45,XY,rob(13;22)(q10;q10)mat	OA*: concentration 2.0; motility <30; normal forms >14	lack of conception
R2	45,XY,rob(13;15)(q10;q10)mat	OA: concentration: 3.2; motility: 26; normal forms: 9	lack of conception
R3	45,XY,rob(13;14)(q10;q10)	OA: concentration: 8.3; motility: 24; normal forms: 5.5	lack of conception
Control	46,XY	N: concentration: 60; motility: 55; normal forms: >14	fertile
WHO[62] guidelines	46,XY	Concentration (10^6/ml): ≥ 15; Progressive motility (%): ≥ 32; Normal forms (%): ≥ 4	

*OA = oligoastenozoospermia; N = normozoospermia.

Supplementary Figure S1. The representative images of NORs localizations (trecognized as FISH signals) according to schemes No. 1 – 23 in Table 6. The nuclei were stained with DAPI, and the NORs are stained with Texas Red. Chromosome centromere 7 was stained with FITC (green) (as the control of FISH efficiency).

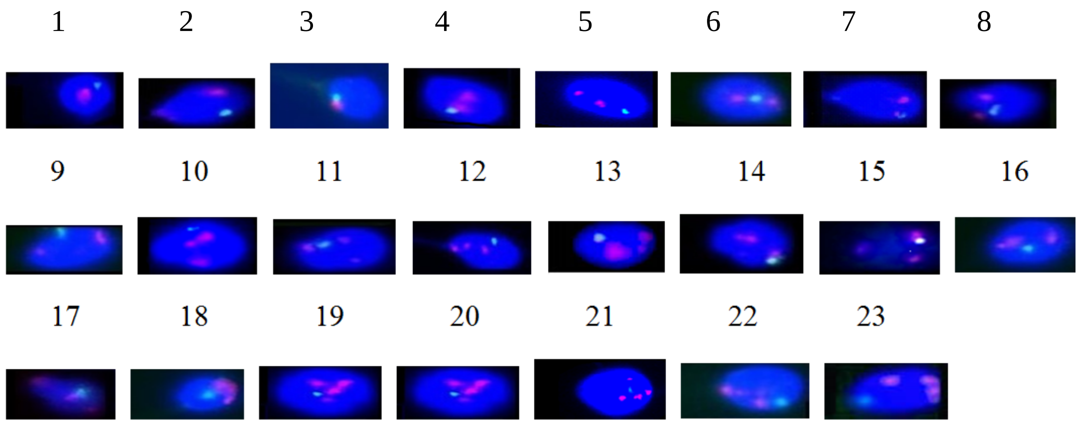

Supplementary Figure S2. Distribution of NORs in the sperm cell nuclei from Robertsonian carriers R1, R2 and R3 and from the Control**. A.** The dispersion (over the nucleus) of NORs (recognized as discrete FISH signals) in all of the analysed sperm cells. **B**. Number of observed discrete NOR signals in the marked radial areas of the sperm nucleus. **C.** Percentage (%) of discrete NOR signals in the marked radial areas (detailed view compared to Figure 2 and Table 7).

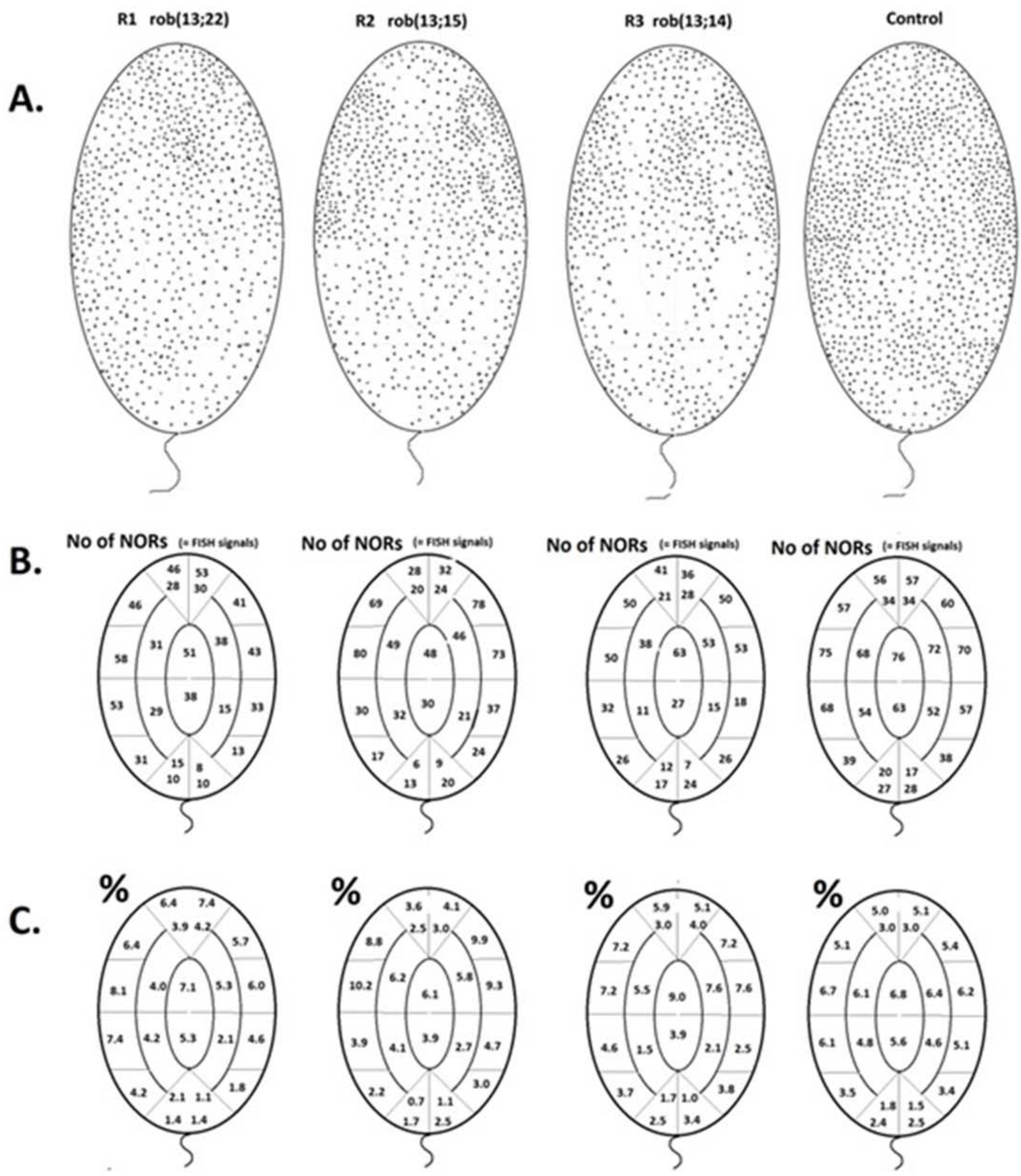

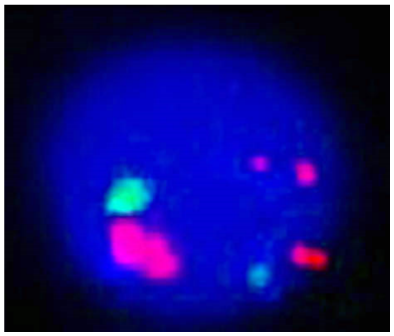

Supplementary Figure S3. Clustering of NORs in a diploid cell (leukocyte) from the control volunteer with normal karyotype . In the representative image of FISH, the nucleus is stained with DAPI and the NORs are stained with Texas Red. Chromosome centromere 7 was stained with FITC (green) (as the control of FISH efficiency).
